# Comparison of Jaw Muscle Activity, Masticatory Performance, and Salivation Across Three Chewing Patterns: A Within‐Subjects Experimental Study

**DOI:** 10.1155/ijod/8230887

**Published:** 2026-08-12

**Authors:** Jarin Paphangkorakit, Sadanun Hongsuwan, Nattanan Rattanasrithong, Benyapatchara Rodma, Rajda Chaichit

**Affiliations:** ^1^ Department of Oral Biomedical Sciences, Faculty of Dentistry, Khon Kaen University, Khon Kaen 40002, Thailand, kku.ac.th; ^2^ Department of Preventive Dentistry, Faculty of Dentistry, Khon Kaen University, Khon Kaen 40002, Thailand, kku.ac.th

**Keywords:** EMG, jaw muscle, mastication, masticatory performance, muscle asymmetry, salivary flow

## Abstract

**Background:**

The effects of simultaneous bilateral chewing are not well understood. Jaw muscle activity, masticatory performance, and salivary production during strictly unilateral, alternate unilateral, and bilateral chewing were investigated.

**Materials and Methods:**

Twenty healthy dental students (aged 20–24 years) with complete natural dentition participated in this repeated‐measures study. Each participant chewed a standardized piece of raw carrot for 20 chewing cycles using three chewing patterns: strictly unilateral on the preferred side, alternate unilateral with side switching every five cycles, and simultaneous bilateral chewing. Masseter and anterior temporalis muscle activity was recorded using surface electromyography. Masticatory performance was assessed by image analysis of the total area of chewed particles, and salivary volume and flow rate were measured gravimetrically. Repeated‐measures ANOVA or Friedman tests were used to compare differences among the three chewing patterns.

**Results:**

Masticatory performance and the masseter asymmetry index (AI) did not differ significantly among chewing patterns. However, the anterior temporalis AI was significantly lower, chewing duration was significantly longer, and subsequently, salivary volume was significantly higher during alternate unilateral and simultaneous bilateral chewing compared with strictly unilateral.

**Conclusion:**

Alternate unilateral and simultaneous bilateral chewing promote more symmetrical jaw muscle activity and prolong chewing time without compromising masticatory performance, suggesting that these patterns may serve as alternative chewing strategies for individuals with strictly unilateral chewing habits.

## 1. Introduction

A balanced strength between the right and left skeletal muscles is one of the requirements for maintaining healthy muscle conditions [1, 2]. Asymmetrical activity in the jaw‐closing muscles is more prevalent in TMD patients [3–5]. Masticatory performance has also been shown to increase in individuals with more symmetrical jaw‐closing muscle activity in the maximum intercuspal position [6], and the symmetrical activity of the jaw‐closing muscles is improved after orthognathic surgery [7].

Chewing in humans usually involves the use of either side of the dentition to break down food. This ensures adequate shearing so that food can be comminuted effectively. A unilateral chewing pattern is unavoidably associated with an imbalanced activity between the jaw muscles on both sides. Therefore, dentists recommend that patients use teeth on both the right and left sides to chew alternately, known as alternate unilateral chewing. Periodic switching of the chewing side has been demonstrated in dentate participants in 25%–30% of chewing cycles. A lower frequency of side switching is associated with males, soft foods, and the final stage of the chewing sequence [8]. Interestingly, switching sides 1, 3, or 5 times during 20 chewing cycles did not appear to alter masticatory performance in young adults having at least 24 natural teeth, whereas chewing time was prolonged with more frequent side switching [9]. Participants perceived greater flavor intensity and salivary flow with 25% side‐switching compared to free‐style or unilateral mastication [10].

Few studies have investigated the effects of simultaneous bilateral chewing. This chewing pattern is characterized by vertical open‐close jaw movements, with the posterior teeth on both sides occluding simultaneously. During the chewing of three natural food items, unidentified chewing cycles with predominantly vertical jaw movements, presumably bilateral chewing, were often observed toward the end of the chewing sequence [11]. This could be associated with the increased dispersion and fragmentation of food particles as chewing progresses. It is speculated that tooth contact during bilateral chewing could improve the jaw muscle symmetry. Although salivary flow was reduced during bilateral chewing of unbreakable artificial blocks [12, 13], this effect has not been investigated using natural foods. In addition, the masticatory performance of bilateral chewing remains unknown. With mostly vertical jaw movements, the performance would presumably be affected since no shearing action occurs. Therefore, the present study primarily aimed to compare jaw muscle asymmetry, masticatory performance, and salivation between strictly unilateral, alternate unilateral and simultaneous bilateral chewing using carrots as the test food. It was hypothesized that bilateral chewing using the teeth on both sides would be associated with more symmetrical jaw muscle activity and increased salivation and would have a minimal effect on masticatory performance.

## 2. Materials and Methods

The present study was approved by the Center for Human Research Ethics at Khon Kaen University (HE6662027; dated 05/03/2023) and registered in the Thai Clinical Trials (TCTR20230605002; dated 05/06/2023). The study utilized a repeated‐measures, within‐subjects experimental design. All participants were informed about the study and provided written informed consent in accordance with the Declaration of Helsinki.

The inclusion criteria required participants to have at least 28 natural teeth with an Angle Class I relationship on both sides, no pain or discomfort during chewing, no current medications that could affect salivation, and no strictly side preference. This preference was assessed using a gum‐chewing test modified from Varela et al. [14]. During the test, participants were asked to pause their chewing after 15, 20, 25, 30, and 35 chews, at which point the examiner visually inspected the location of the chewing gum. Participants who kept the chewing gum on the same side during all five pauses were excluded.

### 2.1. Sample Size Calculation

The sample size was determined based on a pilot study involving 10 participants using the standard algebraic repeated‐measures equation adjusted for within‐subject correlation [15]. With the two‐tailed significance level set at 5% and statistical power at 80%, a sample size of 3–11 participants was required for most primary outcomes (chewing duration, muscle asymmetry index (AI), and salivary volume). For masticatory performance, a clinically significant difference of 100 mm^2^ was employed. This was based on the 675 mm^2^ difference between adjacent categories in a 10‐level scoring of masticatory performance using the gummy jelly chewing test [16], which subsequently required a sample size of 14. For the salivary flow rate, a sample size of 84 was required to detect a maximum variance of 0.40 mL/min.

### 2.2. Study Protocol

All participants were scheduled for the experiments between 4:00 and 6:00 p.m. (before dinner) and asked to refrain from food and drink for at least 1 h before the experiment to reduce the variability in salivation. During the experiment, each participant was seated upright in a quiet, air‐conditioned room inside the dental material research unit. They were asked to chew a piece of raw carrot measuring 1 × 3 × 1 cm (W × L × H) for 20 cycles using three different chewing patterns: strictly unilateral on the preferred side, alternate chewing between the right and left sides every five cycles, and simultaneous bilateral chewing. Specifically, in strictly unilateral chewing, the participant strictly used their preferred side to chew the carrot for 20 cycles. In alternate unilateral chewing, they switched between the right and left sides every five cycles. In simultaneous bilateral chewing, the participant was instructed to use their preferred side to break the carrot in the first chewing cycle and then used both sides of the dentition to chew the carrot particles simultaneously throughout the remaining 19 cycles. Before starting the experiment, all participants were allowed to practice each chewing pattern under supervision until they were familiar with the procedures. Two small, colored sticky pads were attached to the tip of the participant’s nose and chin to visually monitor their jaw movements. Chewing trials in which more than five chewing cycles deviated from the specified pattern were discarded and repeated. Participants were also instructed not to swallow saliva during each chewing trial. Three consecutive trials with 1‐min rest intervals were performed for each chewing pattern, and the order of the patterns was randomized by drawing lots. An additional 2‐min rest was provided between chewing patterns to allow the salivary flow rate to return to baseline. Asymmetry of jaw muscle activity, chewing duration, masticatory performance, total salivary volume, and flow rate (detailed in the following sections) were measured for each trial.

### 2.3. Measurement of Salivary Volume and Salivary Flow Rate

Chewed carrot particles, together with the saliva secreted during chewing, were expectorated into a collecting container. Residual particles remaining in the oral cavity were collected by instructing participants to rinse their mouths with 10 mL of water and expectorate the contents into the same container. The weight of the saliva produced during each chewing trial was determined by subtracting the combined weight of the original unchewed carrot, 10 mL of water, and the empty container from the total weight of the container and its collected contents. To determine salivary density, each participant was subsequently asked to chew a piece of paraffin wax for 5 min, during which saliva was collected. The density of each participant’s saliva was calculated by dividing the weight of the collected saliva by its volume. The individual salivary density was then used to convert salivary weight into salivary volume for each participant. In addition, the salivary flow rate was calculated by dividing the salivary volume by the time spent to complete the 20 chewing cycles.

### 2.4. Measurement of Jaw Muscle Activity

Bilateral masseter (MA) and anterior temporalis (AT) muscle activity was recorded using disposable surface EMG electrodes (Duotrodes Myotronics, Kent, WA) placed on the facial skin, with a ground electrode placed over the fifth cervical vertebra. The electromyographic data were processed through an acquisition system (MP100, BIOPAC Systems, Aero Camino Goleta, CA) utilizing a 1000 Hz sampling rate and a 10–500 Hz bandwidth. Integrated EMG (IEMG) signals during chewing trials and maximum clenching were obtained by rectifying and averaging the signal over increments of 100 data points. These signals were used to calculate the normalized EMG (NEMG) of each muscle to quantify the level of muscle activation. The AI, derived using the absolute value of (NEMG_right_ – NEMG_left_)/(NEMG_right_ + NEMG_left_), was used to quantify any asymmetry in muscle activation between the right and left sides. An AI value of 0 indicated perfect symmetry, whereas a value of 1 represented maximal asymmetry, with muscle activity present on only one side.

### 2.5. Assessment of Masticatory Performance

After salivary volume analysis, the collected carrot particles were rinsed with water and dried using a piece of a paper towel. The particles were then poured onto a transparent polyacrylic plate, measuring 22 × 30 × 2 cm (W × L × H), which was half‐filled with distilled water. The carrot particles were carefully dispersed using a plastic rod to ensure uniform distribution across the plate. The plate was subsequently placed on a digital scanner (HP Deskjet, Ink Advantages 2776, Hewlett–Packard, CA, USA) with a resolution of 300 × 300 dpi. A 10‐mm scale was marked on the lower corner of the bottom of the plate for reference. The scanned image was imported into Image J (National Institutes of Health, Bethesda, Maryland, USA), converted to an 8‐bit black and white image, and analyzed to determine the total area of the particles (mm^2^).

### 2.6. Statistical Analysis

The AI, masticatory performance, chewing duration, salivary flow rate, and salivary volume, averaged across the three trials of each chewing pattern, were used for further statistical analyses. Intraclass correlation coefficients (ICCs) using a two‐way model for absolute agreement of average measures [ICC(2,k)] were calculated to test the consistency of measurements between repeated trials. Shapiro–Wilk tests were used to assess data normality, demonstrating that all study variables, except for masticatory performance and chewing duration, were not normally distributed. Subsequently, descriptive statistics of these variables were reported as medians and interquartile ranges (IQR) for nonnormally distributed variables.

Differences in the outcomes among the three chewing patterns were compared using repeated‐measures ANOVA (for normally distributed variables) or the Friedman test (for nonnormally distributed variables). When a significant main effect was detected, Bonferroni‐adjusted post hoc comparisons were performed (α = 0.0167) using paired *t* or Wilcoxon signed‐rank tests, as appropriate. All analyses were performed using IBM SPSS Version 29, and the significance level was set at *p* < 0.05.

## 3. Results

**Figure 1:**
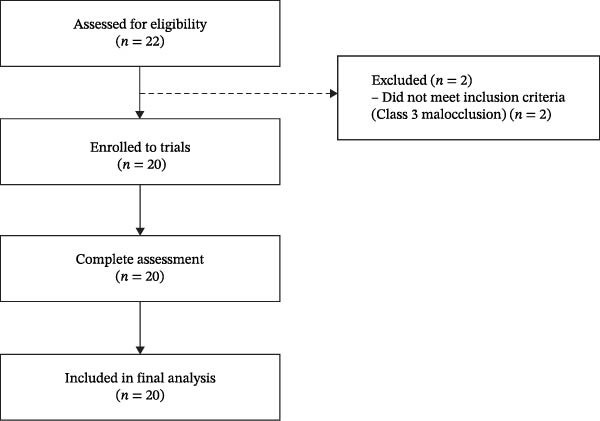
STROBE participant flow diagram.

A total of 22 participants were initially screened for eligibility based on the inclusion and exclusion criteria. Of these, two participants were excluded because of Angle’s Class III malocclusion. Consequently, 20 participants (7 males and 13 females; mean age 23.3 ± 0.5 years) met all criteria and were finally included in the study and subsequent data analysis (Figure 1).

Single‐trial ICCs for masticatory performance were poor‐to‐moderate, ranging from 0.335 to 0.509 across the three chewing patterns. However, averaging the triplicate measurements significantly optimized dataset resilience, yielding acceptable to good reliability coefficients across all groups (strictly unilateral: ICC = 0.712, alternate unilateral: ICC = 0.602, and simultaneous bilateral chewing: ICC = 0.757). ICCs for muscle activity measurements were high, ranging from 0.904 to 0.967 across all muscles, whereas those for salivary flow measurements were moderate, ranging from 0.632 to 0.786 across 3 chewing patterns, but averaging the triplicates achieved good to excellent reliability (0.838–0.917).

Detailed values for the masticatory performance, chewing duration, total salivary volume, salivary flow rate, NEMG (AT and MA), and AI (AT and MA) of each participant are presented in Table S1 (see the Supporting Information).

**Figure 2:**
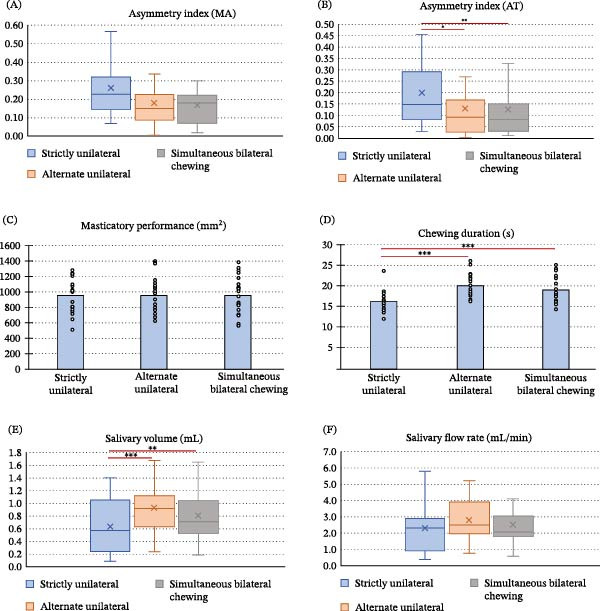
Graphical comparison of (A) the asymmetry index of masseter (MA) muscle activity, (B) the asymmetry index of anterior temporalis (AT) muscle activity, (C) masticatory performance (total area of chewed carrot particles), (D) chewing duration for 20 cycles, (E) total salivary volume, and (F) salivary flow rate during strictly unilateral, alternate unilateral, and simultaneous bilateral chewing. Masticatory performance and chewing duration are presented as means and SDs and subsequently compared using repeated‐measures ANOVA, whereas the remaining variables are presented as medians and interquartile ranges (IQR) and compared using Friedman tests. Bars with asterisks indicate significant post hoc pairwise comparisons after Bonferroni correction. ( ^∗^ *p* < 0.0167;  ^∗∗^ *p* < 0.01;  ^∗∗∗^ *p* < 0.001) (× in box‐and‐whisker plots = means).

There were no significant differences in masticatory performance or AI (MA) among the three chewing patterns. However, AI (AT) was significantly smaller in both alternate unilateral (median: 0.09, IQR: 0.03–0.16; *p* = 0.014) and simultaneous bilateral chewing (median: 0.08, IQR: 0.03–0.13; *p* < 0.01) compared with strictly unilateral (median: 0.15, IQR: 0.09–0.29). In addition, salivary volume was significantly greater in alternate unilateral (median: 0.92 mL, IQR: 0.65–1.10; *p* < 0.001) and simultaneous bilateral chewing (median: 0.71 mL, IQR: 0.53–0.94; *p* = 0.006) compared with strictly unilateral (median: 0.56 mL, IQR: 0.29–0.87). Chewing duration was significantly longer in both alternate unilateral (mean 20.1 ± 2.8 s; *p* < 0.0001) and simultaneous bilateral chewing (mean 19.0 ± 3.1 s; *p* < 0.0001) than in strictly unilateral (mean 16.3 ± 2.5 s). Although there was a significant main effect in the salivary flow rate across the three chewing patterns, post hoc pairwise comparisons could not demonstrate significant differences. There was a trend that the salivary flow rate was increased during alternate unilateral (median: 2.50 mL/min, IQR: 1.98 – 3.75; raw *p* = 0.021 prior to Bonferroni correction) than strictly unilateral (median: 2.29 mL/min, IQR: 0.96–2.87) (Figure 2).

## 4. Discussion

The present study is among the few that have investigated the biology of bilateral chewing. It has been demonstrated that the simultaneous bilateral chewing of carrots promotes more symmetrical jaw muscle activation and increases salivary production through prolonged chewing duration without adversely affecting masticatory performance.

Simultaneous bilateral chewing resulted in a more symmetrical activation of the anterior temporalis but not of the masseter muscles. Activity in the ipsilateral muscles has been shown to be slightly greater than that on the contralateral side during unilateral biting [17, 18]. Such symmetrical muscle activation might be beneficial for the masticatory system, as muscle fatigue is likely to develop in the muscles on the chewing side [19–21] and individuals with a strictly unilateral side preference have been reported to exhibit a higher prevalence of TMD symptoms [22]. A more symmetrical pattern of jaw muscle activity was also observed during alternate unilateral, in which the chewing side was regularly switched.

It was, however, unclear why AI did not differ significantly in the masseter muscle. This finding is in agreement with the findings from a previous study showing that during unilateral clenching, the activity of the masseter on both sides was generally the same, whereas that of the anterior temporalis was greater on the ipsilateral side [23]. The more symmetrical muscle activity is presumed to help maintain the health of the masticatory system, including the jaw muscles and temporomandibular joints.

Longer chewing times were observed during both alternate unilateral and simultaneous bilateral chewing. In alternate unilateral, this was likely due to the additional time required to switch the chewing side. Ignatova‐Mishutina et al. [10] have also demonstrated that chewing time increases when the chewing side is regularly switched. A similar mechanism might apply to bilateral chewing, either because more time is required for the tongue to distribute the bolus to both sides of the dentition or because the jaw‐closing speed was reduced in response to a larger tooth‐food contact area on both sides of the jaw [10]. Additionally, the increased chewing duration was accompanied by a reduced chewing rate. Previous studies have demonstrated that slow chewing is associated with greater energy expenditure, improved detection of foreign material in food, reduced meal intake, and a lower risk of jaw‐closing muscle injury [24–27], all of which are beneficial to the masticatory system.

Masticatory performance was not significantly different among the three chewing patterns based on the clinically significant difference of 100 mm^2^. This finding was consistent with previous studies showing that side switching during the chewing of two‐colored chewing gum [10] or silicone tablets [9] did not affect masticatory performance. It was speculated that unilateral chewing, either strictly or alternating, would improve food shearing due to the lateral component of the chewing stroke as opposed to bilateral chewing. Interestingly, the present study found no difference in masticatory performance between strictly unilateral and simultaneous bilateral chewing. This lack of difference might be because the fragmentation of carrots depends more on crushing than on shearing and therefore does not require substantial lateral jaw excursion.

On the other hand, chewing simultaneously on both sides of the jaw with increased occlusal contact did not appear to enhance masticatory performance compared to unilateral chewing. This might be explained by a reduction in the occlusal force exerted on each individual tooth when a greater number of tooth contacts are engaged during bilateral chewing. The similar levels of jaw‐closing muscle activity observed across the three chewing patterns seem to support the notion that the total resultant bite force was not altered. The unchanged masticatory performance found in the present study could also be due to the fact that dentate participants had readily achieved a similar performance limit within 20 chewing cycles, a so‐called “ceiling effect.” Thus, decreasing the number of cycles might be necessary to improve the discriminating power of the test among dentate participants. However, by employing a threshold of 100 mm^2^, or ~10% of the average performance, as a meaningful difference in the present study, the discriminating ability of the 20‐cycle test could be improved.

The increase in total salivary volume (~35%) observed during alternate unilateral and simultaneous bilateral chewing compared with strictly unilateral was likely the result of the increase in the chewing duration (~20%). This finding aligns with a previous study reporting an 11% increase in perceived salivary flow during 25% side‐switching mastication compared to unilateral chewing [10]. Because the present study was not explicitly powered to detect subtle variances in the salivary flow rate, we cannot definitively conclude whether the increased volume was solely dependent on prolonged chewing. However, an observed trend toward an increased salivary flow rate during alternate unilateral (*p* = 0.021; failing to reach statistical significance after Bonferroni correction, adjusted α = 0.0167), together with an unequal percentage increase between the salivary volume and chewing duration, suggests that the increased salivary flow rate might also partially contribute to the greater overall fluid volume.

The present study has several limitations. First, the findings from only one food type might not be generalized. Other food types, such as meat or tougher diets, might not be effectively fragmented during bilateral chewing. However, given that a combination of food textures is routinely consumed, bilateral chewing may still be advisable, particularly during the late stage of the chewing sequence when food is sufficiently softened [11]. Second, although participants were allowed to practice each chewing pattern well before the trials, their actual chewing may not have been perfect, particularly when they were unfamiliar with chewing on their nonpreferred side. However, the relatively small effect on masticatory performance and the clear differences in the AI across the three chewing patterns indicated a true alteration of the chewing pattern. Thirdly, although an observed trend suggested an increased salivary flow rate during alternate unilateral compared to strictly unilateral, the study was underpowered to achieve statistical significance. Consequently, this limited sample size cannot confirm whether the lack of detectable difference between simultaneous bilateral chewing and strictly unilateral chewing is a true null effect. Lastly, it should be noted that there could be some carryover effects of salivation and jaw muscle activity from trial to trial. According to Anderson et al. [12], the stimulated salivary flow rate in the parotid gland returns to baseline within 30 s after chewing gum. Given the mild taste of the chewed carrots, it is highly likely that any salivary carryover effect was minimized by the 1‐min rest between trials of the same chewing pattern and the 2‐min rest between trials of different chewing patterns. Furthermore, it is unlikely that muscle fatigue developed during the experiment since the jaw‐closing muscles have been shown to be fatigue‐resistant, requiring ~500 s of continuous chewing to experience fatigue [28].

In conclusion, the present study has demonstrated that alternate unilateral and simultaneous bilateral chewing of food items similar to carrots increases the chewing duration and promotes symmetrical jaw muscle activity without compromising masticatory performance. The longer chewing duration subsequently increases the total salivary volume. This suggests that both may be an alternative chewing practice for individuals who are strictly unilateral chewers.

## 5. Conclusion

Using carrots as the test food in this controlled clinical study, both alternate unilateral and simultaneous bilateral chewing enhanced symmetrical jaw muscle activity, increased chewing duration, and subsequently resulted in a greater salivary volume compared with strictly unilateral chewing, without compromising masticatory performance. Both chewing patterns could be beneficial for the health of the masticatory system and may be used as a rehabilitative strategy for individuals with strictly unilateral chewing habits.

## Author Contributions

**Jarin Paphangkorakit**: writing – original draft, review and editing, conceptualization, supervision, methodology, investigation, data curation. **Sadanun Hongsuwan, Nattanan Rattanasrithong, and Benyapatchara Rodma**: writing – review and editing, methodology, investigation. **Rajda Chaichit**: writing – review and editing, methodology, formal analysis.

## Funding

The present study was supported by the Faculty Research Grant, Faculty of Dentistry, Khon Kaen University.

## Disclosure

All authors have read and approved the final version of the manuscript. Jarin Paphangkorakit had full access to all of the data in this study and takes complete responsibility for the integrity of the data and the accuracy of the data analysis.

## Conflicts of Interest

The authors declare no conflicts of interest.

## Supporting Information

Additional supporting information can be found online in the Supporting Information section.

## Supporting information


**Supporting Information** Table S1: Masticatory performance, chewing duration, preferred chewing side, total salivary volume, salivary flow rate, muscle activity (NEMG), and asymmetry index of jaw muscle activity during strictly unilateral, alternate unilateral, and simultaneous bilateral chewing of each individual participant. (MA = masseter, AT = anterior temporalis, R = right, L= left). Means, standard deviations (STD), medians, interquartile ranges (IQR) are also shown.

## Data Availability

The authors confirm that the data supporting the findings of this study are available within the article and/or its supporting information.
